# Technetium Encapsulation by A Nanoporous Complex Oxide 12CaO•7Al_2_O_3_ (C12A7)

**DOI:** 10.3390/nano9060816

**Published:** 2019-05-30

**Authors:** Navaratnarajah Kuganathan, Alexander Chroneos

**Affiliations:** 1 Department of Materials, Imperial College London, London SW7 2AZ, UK; alexander.chroneos@imperial.ac.uk; 2 Faculty of Engineering, Environment and Computing, Coventry University, Priory Street, Coventry CV1 5FB, UK

**Keywords:** technetium, DFT, encapsulation, C12A7, electride

## Abstract

Technetium (^99^Tc) is an important long-lived radionuclide released from various activities including nuclear waste processing, nuclear accidents and atmospheric nuclear weapon testing. The removal of ^99^Tc from the environment is a challenging task, and chemical capture by stable ceramic host systems is an efficient strategy to minimise the hazard. Here we use density functional theory with dispersion correction (DFT+D) to examine the capability of the porous inorganic framework material C12A7 that can be used as a filter material in different places such as industries and nuclear power stations to encapsulate Tc in the form of atoms and dimers. The present study shows that both the stoichiometric and electride forms of C12A7 strongly encapsulate a single Tc atom. The electride form exhibits a significant enhancement in the encapsulation. Although the second Tc encapsulation is also energetically favourable in both forms, the two Tc atoms prefer to aggregate, forming a dimer.

## 1. Introduction

Long-lived Technetium (^99^Tc) is a dominant radionuclide which is mainly introduced into the environment due to human activities such as nuclear weapon testing, nuclear spent fuel reprocessing and radio-pharmaceutical activities [1,2,3,4]. Importantly, technetium has radioactivity with a long half-life (2.1 × 10^5^ years), and there is a health risk when it enters the human body [4,5]. Thus, the effective capture of Tc from the environment is of great interest.

There has been an extensive search for candidate host materials to trap radionuclides [6,7,8,9]. The requirements imposed on host materials are high thermodynamical, chemical, and mechanical stability. A number of host materials including zeolites, buckyballs (C_60_), carbon nanotubes and metal organic frameworks have been studied extensively [10,11,12,13,14]. The buckyball structured fullerenes have been recently considered as candidate host materials for the encapsulation or trapping of many species, including radionuclides, as they have open structures with very high mechanical stability at high temperatures and are chemically inert [15,16,17,18,19,20]. Saha et al. [21] applied a recoil implantation technique to encapsulate a variety of radio isotopes for future application in radioactive endohedral fullerenes. Endohedral encapsulation of single Tc atoms and Tc clusters was studied theoretically to predict the structures and magnetic properties of the resultant complexes by Weck et al. [22]. Silica zeolite was effectively used by Pham et al. [23] to capture radioactive iodine, which directly affects the human metabolic system. Peng et al. [24] proposed a new strategy to trap radioactive barium and other nuclides using a metal organic framework. The search for cheaper and “greener” alternative stable host materials continues.

**Figure 1:**
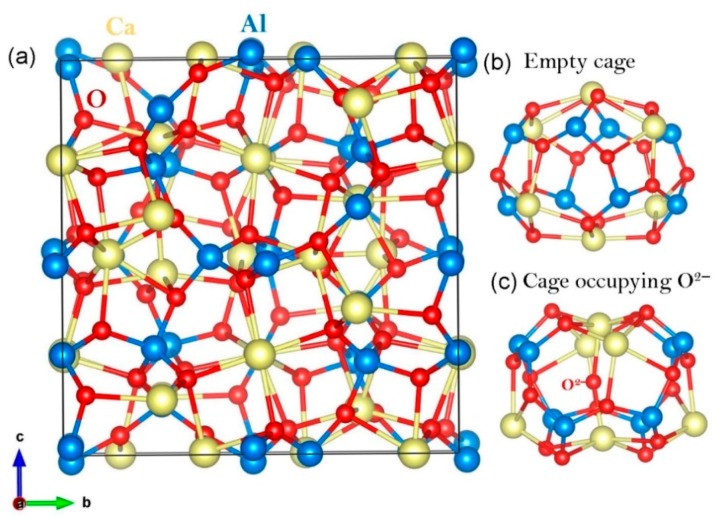
(**a**) Crystal structure of [Ca_24_Al_28_O_64_]^4+^ containing twelve empty cages; (**b**) and (**c**) optimised structures of an empty cage and a cage with an extra-framework O^2−^ ion in C12A7:O^2−^, respectively.

12CaO•7Al_2_O_3_ (C12A7) is a complex inorganic oxide exhibiting high thermal and chemical stability [25] (refer to Figure 1). The stability of this material under harsh conditions and its powder form are beneficial to preparing filters in experiments and capturing Tc efficiently. Its unusual crystal structure consists of 12 nano cages per unit cell, making this material promising for accommodating a large number of foreign species such as atoms or small molecules [25,26]. Additionally, its constituent elements are non-toxic and highly abundant. The cubic unit cell of stoichiometric C12A7 is represented by the formula [Ca_24_Al_28_O_64_]^4+^·2O^2−^ (C12A7:O^2−^) [25,26], in which the cation [Ca_24_Al_28_O_64_]^4+^ makes up the framework of 12 equivalent cages, each having a mean effective charge of 1/3 |e| and connected to eight neighbouring cages via ~0.1 nm wide openings. The two O^2−^ ions compensate the positive charge of the framework. They occupy one in six cages and are often referred to as extra-framework or “free” anions. The extra-framework O^2–^ ions are relatively loosely bound to the framework and can be either removed or replaced with other species, such as H^–^, OH^−^, Au^−^, NH_2_^−^, F^−^, and others [27,28,29,30,31,32,33,34]. Complete replacement of two extra-framework O^2−^ ions can also occur with four electrons by effective reduction methods. This form of C12A7 is regarded as an electride, i.e., a material in which electrons act as anions, and is denoted as C12A7:*e*^−^ [35,36].

In the present study, density functional theory with dispersion correction (DFT+D) is used to examine the thermodynamic stability of gas-phase Tc atoms and a Tc dimer encapsulated within C12A7 by calculating the encapsulation energies, Bader charges on encapsulated atoms, density of states (DOS) plots and charge density plots. This study can provide useful information about the practical applicability of this material in filters used in many places, including nuclear reprocessing plants and pharmaceutical industries. Furthermore, the application of this material can be extended to the efficient capture of Tc released into the environment during unexpected nuclear accidents. DFT calculations provide valuable information on equilibrium structures, charge transfer and electronic properties.

## 2. Computational Methods

We used DFT together with spin polarisation as implemented in the Vienna Ab initio Simulation Package (VASP) code [37] to calculate the electronic structures of Tc atoms and a Tc dimer encapsulated within C12A7 nanopores. The DFT method is powerful tool to model periodic systems (e.g., C12A7) and provide accurate information on structures, electronic structures, charge transfer and weak intermolecular forces. In previous studies, we used DFT methods to model a variety of species encapsulated in different host materials [38,39,40,41,42,43,44,45,46,47,48,49,50,51]. There were 118 and 116 atoms present in the stoichiometric and electride forms of the cubic supercell, respectively. Generalised gradient approximation (GGA) parameterised by Perdew, Burke, and Ernzerhof (PBE) was used to describe the exchange–correlation term [52]. A plane wave basis set of 500 eV and a 2 × 2 × 2 Monkhorst–Pack *k*-point mesh [53] were used. This combination was used in previous studies, and further increase in the cut-off energy or *k* resulted in negligible change in the total energy [32]. For bulk Tc, an 8 × 8 × 6 Monkhorst–Pack *k*-point mesh was used. Geometry optimisations were carried out using a conjugate gradient algorithm [54]. Forces on the atoms were calculated using the Hellman–Feynmann theorem; the Pulay corrections were smaller than 0.001 eV/Å, and the atomic stress tensor values were less than 0.002 GPa. The encapsulation energy for a single Tc atom inside an empty cage of the electride form of C12A7 is defined by the following equation:

E_Enc_ = E(Tc:C12A7:e^−^) − E(C12A7:e^−^) − E(Tc) (1)

where E (C12A7:e^−^) is the total energy for bulk C12A7:e^−^, E (Tc:C12A7:e^−^) is the total energy of the Tc atom occupying the cages, and E(Tc) is the total energy of an isolated Tc atom (the reference state).

Here, semi-empirical dispersion corrections were included using the pair-wise force field as implemented by Grimme et al. [55] [DFT-D3 (zero)] in VASP.

## 3. Results

### 3.1. Structural Modelling of Bulk Tc and C12A7

First, the validity of the pseudopotentials and basis sets was examined by performing energy minimisation calculations on bulk Tc and bulk C12A7 and comparing the equilibrium lattice constants with corresponding experimental values.

Hexagonal bulk Tc (space group P63/mmc) was optimised under constant pressure to validate the pseudopotential and basis set used for Tc. The calculated lattice constants are in good agreement with the experimental values reported by Lam et al. [56]. Table 1 reports the comparison of the lattice constant values obtained by the experiment and this study.

The cubic unit cell of stoichiometric C12A7 (space group $\left(I\bar{4}3d\right)$) has a lattice constant of 11.99 Å [26]. The experimental structure of C12A7:O^2−^ was optimised to validate the pseudopotentials and basis sets used for Ca, Al and O. There is a good reproduction between the calculated and experimental lattice constants as observed in our previous study [32] (refer to Table 2). There is a small distortion in the cubic symmetry due to the attraction between the extra-framework O^2−^ and the cage wall Ca^2+^ ions introducing a small perturbation in the lattice parameters (refer to Figure 1). Nevertheless, this distortion is small and can be deemed acceptable.

### 3.2. Encapsulation of Single Tc Atoms in A Cage of C12A7:O^2−^

**Figure 2:**
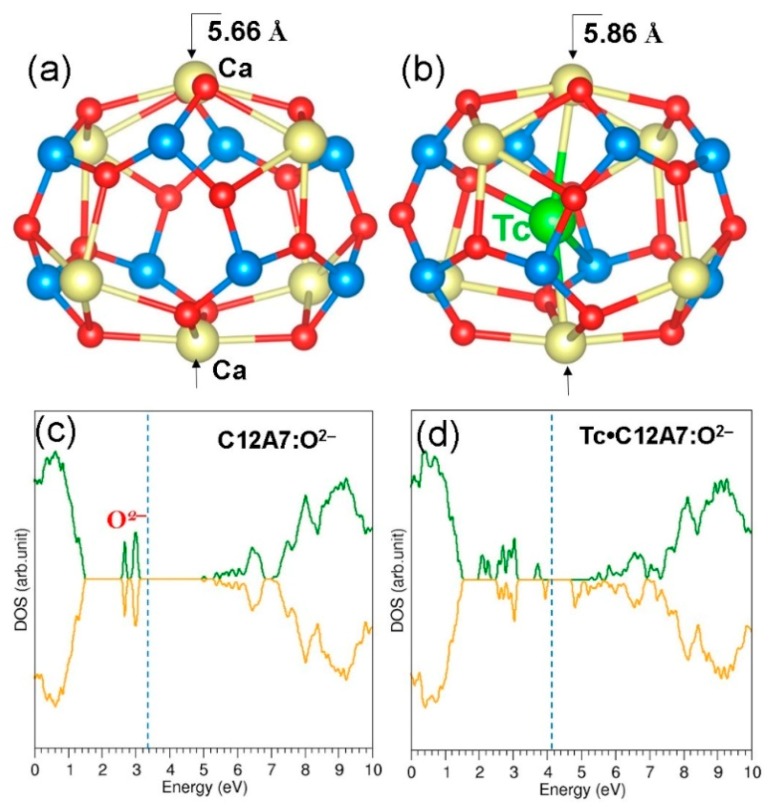
(**a**) and (**b**) Optimised structures of an empty cage and a cage containing a Tc atom, respectively, showing the Ca–Ca cage pole distance; (**c**) and (**d**) density of states (DOS) plots calculated for C12A7:O^2−^ and Tc:C12A7:O^2−^, respectively. The vertical blue dotted lines correspond to the Fermi energy.

The thermodynamic stability of a single Tc atom encapsulated within one of the ten empty cages in stoichiometic C12A7 was examined. Figure 2 shows the relaxed structure and the cage containing the Tc atom. Tc occupies a position slightly off centre in the cage, forming strong Tc–O bonds with bond distances of 2.33 Å and 2.54 Å. Nevertheless, the Tc–Ca and Tc–Al distances are relatively long (Tc–Ca: 2.87 Å and 3.14 Å, Tc–Al: 2.97 Å) due to the repulsion between cations. This is reflected in the Ca–Ca cage pole distance (5.86 Å). There is a 0.2 Å elongation in the Ca–Ca cage pole distance compared to that of the empty cage (refer to Figure 2). The encapsulation energy is −2.24 eV, suggesting that a gaseous Tc atom is more stable inside the cage than its isolated form. The Bader charge analysis [57] result on the Tc is +0.13. This small Bader charge is due to the net interactions between Tc and cations and anions. The overall structure of the lattice was not significantly affected by the encapsulation of a single Tc. Figure 2c,d show the DOS plots for C12A7:O^2−^ and Tc encapsulated within C12A7:O^2−^ (Tc: C12A7:O^2−^), respectively. C12A7:O^2−^ is an insulator with a wide band gap. Encapsulation of Tc introduces gap states closer to the Fermi level, and the resultant complex is still an insulator.

**Figure 3:**
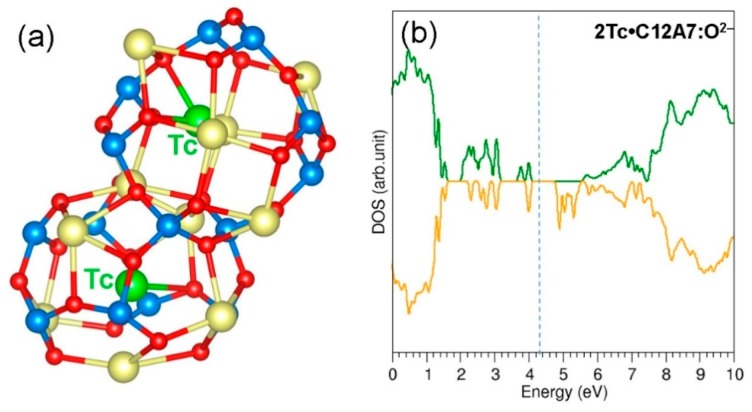
(**a**) Relaxed cages containing two Tc atoms in C12A7:O^2−^ and (**b**) DOS plot calculated for 2Tc: C12A7:O^2−^.

Thereafter, we considered an additional Tc atom in a cage adjacent to the cage with a pre-existing Tc. The relaxed cages are shown in Figure 3a. The Ca–Ca cage pole distances were calculated to be 5.85 and 6.00 Å, again showing the repulsion between Tc and cage pole Ca^2+^ ions. Tc atoms are displaced slightly away from the centre of the cages and interact with cage wall ions. The calculated Tc–O bond distances were 2.24 Å and 2.74 Å. The encapsulation energy for the second Tc atom into the Tc:C12A7:O^2−^ is −2.36 eV, suggesting that further incorporation of Tc into the empty cages is energetically feasible. The second incorporation energy is slightly more negative (by 0.12 eV) than the first incorporation energy. This is due to the small expansion of volume introduced by the previous geometry optimisation for Tc:C12A7:O^2−^. The Bader analysis shows that both Tc atoms exhibit a +0.13 charge. The DOS plot is shown in Figure 3b. Additional states are introduced in the band gap by the second Tc atom. The introduction of Tc atoms into the cages leaves the composite magnetic according to the DOS plot as the spin-up and spin-down states are not equal, particularly in the band gap region.

### 3.3. Encapsulation of Single Tc Atoms in C12A7:e^−^

**Figure 4:**
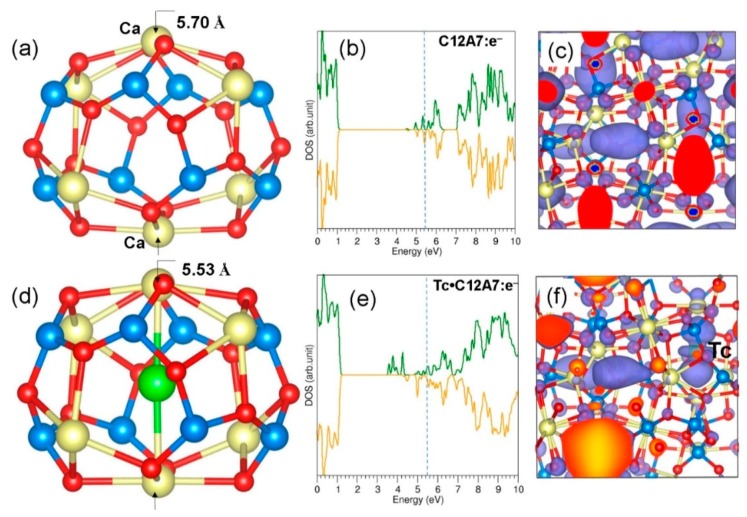
(**a**) Relaxed empty cage showing the Ca–Ca pole distance in C12A7:e^−^; (**b**) DOS plot calculated for C12A7:e^−^; (**c**) charge density plot showing four electrons delocalised in C12A7:e^−^; (**d**) relaxed cage containing Tc; (**e**) DOS plot calculated for Tc: C12A7:e^−^; and (**f**) charge density plot showing the localisation of some electrons on the Tc atom.

The electride form of C12A7 was next considered for the encapsulation of a single Tc atom. Figure 4d shows the optimised structure of the cage with encapsulated Tc. In order to compare the deformation, the optimised structure of an empty cage in C12A7:e^−^ is also shown in Figure 4a. The encapsulated Tc atom occupies a space between two Ca^2+^ ions in the cage wall (center of the cage). This is due to the strong attraction between Tc and the positively charged cage wall Ca^2+^ ions. This is further supported by the Bader charge on the Tc (−0.90 |e|). The negative charge on the Tc atom was transferred from the extra framework electrons available in the electride form of C12A7. The cage wall distance (refer to Figure 4d) is 5.53 Å. This is shorter by 0.17 Å than that calculated for an empty cage (refer to Figure 4a). This is due to the electrostatic attraction between the negatively charged Tc and positively charged cage wall Ca^2+^ ions. The encapsulation energy was calculated to be −3.56 eV. This is more negative by ~1.30 eV than that calculated for single Tc encapsulation within C12A7:O^2−^. This significant enhancement in the encapsulation energy is due to the delocalised electrons in C12A7:e^−^. These electrons enabled Tc to gain a small number of electrons to form a negative ion which introduces an attractive interaction with cage wall Ca^2+^ ions. Figure 4b,e show the DOS plots for C12A7:e^−^ and Tc:C12A7:e^−^, respectively. C12A7:e^−^ is metallic. The encapsulation of Tc reduces the number of electrons in C12A7:e^−^ and forms a Tc^−^ ion. The resultant complex is still metallic (refer to Figure 4e) as there are three electrons available in the unit cell of C12A7:e^−^. In order to confirm the electron transfer from C12A7:e^−^ to Tc, we plotted constant charge density plots for both C12A7:e^−^ and Tc:C12A7:e^−^ (refer to Figure 4c,f). There is a reduction in the electron charge density in the empty cages of Tc:C12A7:e^−^, and there is an electron cloud localised on the Tc atom, ensuring the electron transfer.

**Figure 5:**
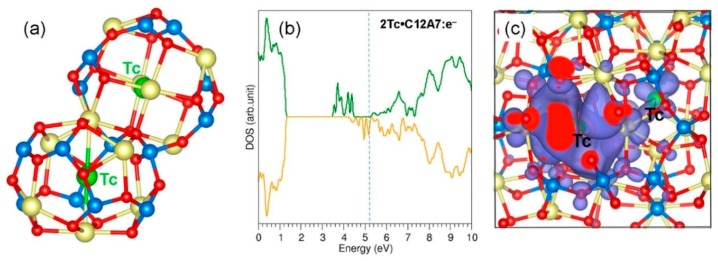
(**a**) Relaxed structure of two Tc atoms encapsulated in two adjacent cages in C12A7:e^−^; (**b**) DOS plot calculated for 2Tc: C12A7:e^−^; and (**c**) charge density plot showing the distribution of electrons in the lattice.

The second Tc atom was next encapsulated within a cage adjacent to the cage with a pre-existing Tc. The relaxed structure of the cages containing Tc atoms is shown in Figure 5a. Both Tc atoms occupy the centre of the cages. The cage pole distances are 5.52 Å and 5.63 Å, showing the attractive interaction between Tc and Ca^2+^ ions. The Bader charges on the Tc atoms are −0.81 |e| and −0.93 |e|. This indicates that the second Tc atom also gains ~1 electron from the extra-framework electrons. There is a reduction in the encapsulation energy (−2.53 eV) for the second Tc. This is due to the lesser number of electrons (three electrons) available in the Tc:C12A7:e^−^ complex. Figure 5b shows the DOS plot calculated for 2Tc: C12A7:e^−^. The resultant complex is metallic as there are two extra-framework electrons available in the lattice. The charge density plot is shown in Figure 5c. Both Tc atoms are surrounded by electrons. The rest of the electrons are localised closer to the cages occupied by the Tc atoms.

### 3.4. Stability of a Tc Dimer in C12A7:O^2−^

**Figure 6:**
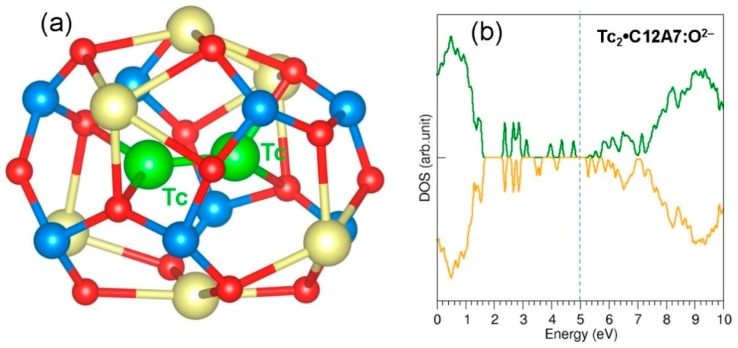
(**a**) Relaxed cage structure showing the accommodation of the Tc dimer in C12A7:O^2−^ and (**b**) DOS plot calculated for Tc_2_: C12A7:O^2−^.

Here we examine the formation of a Tc dimer in an empty cage of C12A7:O^2−^. The relaxed structure of the Tc dimer encapsulated within an empty cage is shown in Figure 6a. The Tc–Tc bond distance is 1.95 Å. This ensures the formation of a dimer. The encapsulation energy of the dimer was calculated according to three different processes (refer to Table 3). In the first process, two gas-phase Tc atoms are encapsulated by C12A7:O^2−^ to form a dimer. The encapsulation energy is −3.78 eV. The formation of the Tc dimer was considered to be due to the reaction between a single Tc atom and Tc:C12A7:O^2−^ in the second process. In this process, the encapsulation energy is −2.65 eV. The lower encapsulation energy is due to the energy required to reorient the pre-existing Tc to form the dimer. In the third process, the encapsulation was considered to be of a gas-phase Tc dimer. The encapsulation energy (−1.16 eV) is still exoergic but is lower than the values calculated in the other processes. This is due to the longer Tc–Tc bond distance by 0.11 Å than that calculated for the gas-phase Tc dimer. The total energy difference between a Tc dimer in an empty cage and two Tc atoms in two adjacent C12A7:O^2−^ cages was calculated. A Tc dimer in an empty cage is more stable (by 2.96 eV) than the two isolated Tc atoms occupying adjacent cages. The Bader charges on the Tc atoms are −0.44 and +0.33, indicating that the dimer is polarised. The relaxed structure of the cage is slightly distorted due to the attractive (between the positively charged Tc and cage wall O^2−^ ions) and repulsive (negatively charged Tc and cage wall O^2−^ ions) forces.

### 3.5. Stability of a Tc Dimer in C12A7:e^−^

**Figure 7:**
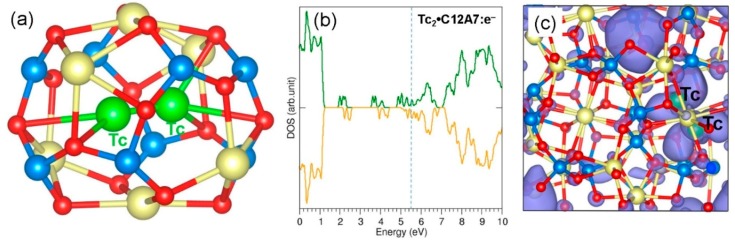
(**a**) Relaxed cage structure showing the Tc dimer in C12A7:e^−^; (**b**) DOS plot calculated for Tc_2_:C12A7:e^−^; and (**c**) charge density plot associated with the electron distribution.

Finally, we considered the stability of a Tc dimer in an empty cage in C12A7:e^−^. The optimised structure of the dimer inside the cage is shown in Figure 7a. The dimer distance is 2.04 Å, slightly longer (by 0.2 Å) than the gas-phase dimer distance. The elongation is due to the repulsion between the two negatively charged Tc atoms. The Bader charges on the Tc atoms are −0.52 |e| and −0.38 |e|, suggesting that the dimer is reduced to form a (Tc_2_)^−^ ion (refer to Table 4). The Tc–O distances (2.52 Å, 2.87 Å, and 2.98 Å) are longer than the values calculated in C12A7:O^2−^ as there is a repulsion between the Tc and cage wall O^2−^ ions. The encapsulation energy for dimer formation from two Tc gas-phase atoms is −3.93 eV (refer to Table 4). The encapsulation of a dimer via this route is more easily facilitated (by 0.16 eV) than that observed in C12A7:O^2−^. The encapsulation energy for a single gas-phase Tc atom to combine with Tc:C12A7:e^−^ to form a dimer is −2.15 eV. As discussed earlier, this is due to the energy required to reorient the pre-existing Tc atom in the cage. There is a reduction in the encapsulation energy by 0.50 eV compared to the similar process in C12A7:O^2−^. This is due to the stronger attraction between the negatively charged Tc atom and the cage wall Ca^2+^ ions. The dimer configuration is 1.77 eV more stable than the two isolated Tc atoms occupying adjacent cages. The encapsulation energy calculated using the dimer as a reference is −1.31 eV, slightly (by 0.15 eV) more favourable than that observed in C12A7:O^2−^.

## 4. Conclusions

We used DFT with dispersion correction to study the encapsulation of gas-phase Tc atoms and dimers into the stoichiometric and electride forms of C12A7. Both forms of C12A7 strongly encapsulate a single Tc atom, and their encapsulation energies are −2.26 eV and −3.56 eV, respectively. These exoergic encapsulation energies suggest that a single Tc atom is more favourable inside the cages of both forms of C12A7 than its gas phase. There is a significant enhancement in the encapsulation energy for C12A7:e^−^ due to the extra-framework electrons being reflected in the Bader charges. The second encapsulation is also exoergic in both cases, suggesting that this material has the ability to encapsulate multiple atoms. The encapsulation energies for the formation of a dimer via encapsulation of two gas-phase Tc atoms in C12A7:O^2−^ and C12A7:e^−^ are −3.78 eV and −3.93 eV, respectively. The similar encapsulation of a dimer as a reference is less favoured than the former. Our study further suggests that two isolated Tc atoms in adjacent cages would prefer to form a dimer in both C12A7:O^2−^ and C12A7:e^−^. The maximum number of Tc atoms with exoergic encapsulation should be our interest in future work.

## Figures and Tables

**Table 1:** Comparison between the experimental and calculated structural parameters of hexagonal (P63/mmc) Tc.

Parameter	Calcd	Exptl [56]	|∆|(%)
a = b (Å)	2.723	2.743	0.73
c (Å)	4.378	4.400	0.50
α = β	90.00	90.00	0.00
γ (°)	120.0	120.0	0.00
V (Å^3^)	28.26	28.67	1.43

**Table 2:** Comparison between the experimental and calculated structural parameters of cubic $(I\bar{4}3d$) C12A7:O^2−^.

Parameter	Calcd	Exptl [26]	|∆|(%)
a (Å)	12.04	11.99	0.42
b (Å)	12.01	11.99	0.17
c (Å)	12.01	11.99	0.17
α (°)	90.02	90.0	0.02
β (°)	89.95	90.0	0.06
γ (°)	89.93	90.0	0.08
V (Å^3^)	1738.66	1727.38	0.65

**Table 3:** Encapsulation energy and Bader charge calculated for the formation of a Tc dimer inside C12A7:O^2−^.

Reaction	Encapsulation Energy (eV/atom) with Respect to Tc Atom	Bader Charge |e| on Tc
Tc + Tc + C12A7:O^2−^ → Tc_2_: C12A7:O^2−^	−3.78	+0.33, −0.44
Tc + Tc: C12A7:O^2−^ → Tc_2_: C12A7:O^2−^	−2.65	
	with respect to Tc dimer (eV/atom)	
Tc_2_ + C12A7:O^2−^ → Tc_2_: C12A7:O^2−^	−1.16	

**Table 4:** Encapsulation energy and Bader charge calculated for the formation of the Tc dimer inside C12A7:e^−^.

Reaction	Encapsulation Energy (eV/atom) with Respect to Tc Atom	Bader Charge |e| on Tc
Tc + Tc + C12A7:e^−^ → Tc_2_: C12A7:e^−^	−3.93	−0.38, −0.51
Tc + Tc: C12A7:e^−^ → Tc_2_: C12A7:e^−^	−2.15	
	with respect to Tc dimer (eV/atom)	
Tc_2_ + C12A7:e^−^ → Tc_2_: C12A7:e^−^	−1.31	
